# Cytophagic Histiocytic Panniculitis (CHP) in a Patient with SLE Found after Autopsy: When a Rash Is “Complicated!”

**DOI:** 10.1155/2019/6830862

**Published:** 2019-07-03

**Authors:** Hafsa Abbas, Ahsan Aslam, Muhammad Saad, Masooma Niazi, Sridhar Chilimuri

**Affiliations:** ^1^Department of Medicine, Division of Gastroenterology, Bronxcare Hospital Center, Bronx, NY 10457, USA; ^2^Department of Medicine, IU Health University Hospital, Indianapolis, IN 46202, USA; ^3^Department of Medicine, Bronxcare Hospital Center, Bronx, NY 10457, USA; ^4^Department of Pathology, Bronxcare Hospital Center, Bronx, NY 10457, USA

## Abstract

**Introduction:**

Cytophagic histolytic panniculitis (CHP) is a clinical disorder characterized by nodular panniculitis of the subcutaneous adipose tissue. It was first described in 1980 by Winkelmann. Histologically it is described as an infiltration of the adipose tissue by T- lymphocytes and phagocytic macrophages (also known as “bean bag cells”). Most of the cases are reported under the age of 50 and is a rare cause of panniculitis. We report a case of CHP in a young patient who presented to our emergency room (ER).

**Case Summary:**

A 39-year-old African American woman who presented to our hospital with lethargy, progressive confusion, and generalized rash involving both lower extremities of 1 week duration. She had a history of pancytopenia and focal proliferative and membranous lupus nephritis classes 3 and 5. Her physical examination was remarkable for bilateral lower extremity pitting edema and a desquamating rash on both of her legs. The Nicolsky sign was positive. She was noted to be hypotensive and was started on intravenous fluids and broad spectrum antibiotics. Routine laboratory tests revealed severe pancytopenia, with a hemoglobin of 3.9 g/dl, white blood cell count 600/ul, and platelet count of 11000/ul. Within an hour of arrival to the ER she developed acute respiratory failure. She was intubated and placed on mechanical ventilation. She developed shock requiring vasopressors. No imaging could be done due to her unstable condition. Four hours after her initial presentation she developed asystole and expired. Postmortem histopathology of the adipose tissue revealed CHP.

**Conclusion:**

CHP can be rapidly fatal. The treatment involves high dose of intravenous steroids and immunosuppressants such as cyclosporine.

## 1. Introduction

Cytophagic histiocytic panniculitis (CHP) is a rare clinical disorder, characterized by nodular panniculitis of the subcutaneous adipose tissue [1]. It was first described in 1980 by Winkelmann, as an infiltration of the subcutaneous adipose tissue by T lymphocytes and phagocytic histiocytes (also known as the “bean bag cells”) [2]. It is a rare cause of aseptic panniculitis [2–4] that is seen in association with viral infections (HSV, EBV) [5], hematopoietic disorders (malignant lymphoma), hemophagocytic lymphohistiocytosis (HLH), acute leukemias (acute lymphoid leukemia, acute myeloid leukemia), Hodgkin's and non-Hodgkin's lymphoma, rhabdomyosarcoma, neuroblastoma, and Langerhans cell histiocytosis [6, 7]. Reports of CHP after H1N1 vaccination are available in the literature [8]. Its association with autoimmune diseases like systemic lupus erythematosus (SLE) is also reported and has a high mortality rate [9–12]. However, only a few such cases confirming the association between SLE and CHP are available. We report one such case of CHP in a patient with SLE that ultimately resulted in the demise of the patient.

## 2. Case Report

A 39-year-old African American woman was brought to our emergency room (ER) with lethargy, progressive confusion, and generalized rash involving both lower extremities of 1-week duration. Two months ago, the patient had presented to our hospital with left lower quadrant pain and nonbloody diarrhea and dizziness. A Computed Tomography (CT) of the abdomen and pelvis had revealed pancolitis and she was treated with antibiotics. At that time she was also found to have proteinuria, pedal edema and photosensitive rash on her face. The proteinuria was attributed to glomerular disease of unclear etiology. Autoimmune work-up revealed positive ANA, anti-Smith Ab, and anti-RNP. Parvovirus IgG was also positive. She was found to have pancytopenia and the diagnosis of aplastic anemia was considered and she was transferred to another tertiary care hospital. There, she underwent a renal biopsy that revealed focal proliferative and membranous lupus nephritis classes 3 and 5. She was discharged on prednisone, mycophenolate, and hydroxychloroquine. Now she had presented with the current complains.

**Figure 1:**
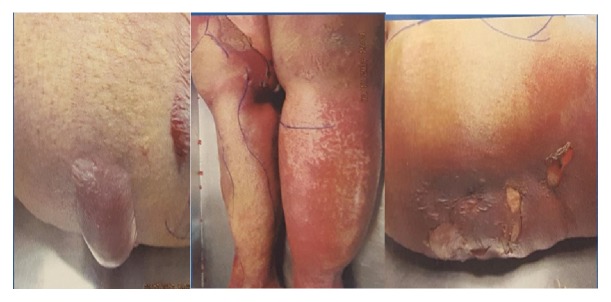
Rash on the back with bullae, rash on the posterior aspect of legs, and desquamating rash on the back.

In the ER she was found to be lethargic. On physical examination, her temperature was 97.5°F, pulse was 102 beats per minute, the initial blood pressure was 136/79 mm of Hg, and respiratory rate was 22 breaths per minute. There was no scleral icterus. Oral mucosa was dry without visible lesions. The neck was supple. Skin was warm and had desquamating rash on both lower extremities from hip down (Figure 1). The rash was nonblanching and erythematous, and Nicolsky sign was positive. The abdomen was soft but mild tenderness was noted in the epigastric region without any guarding or rebound tenderness. There was no organomegaly and the bowel sounds were sluggish. There was bilateral pitting pedal edema. The patient was arousable with verbal and tactile stimulation and was moving all extremities spontaneously. Rest of the physical examination was unremarkable.

Later she developed hypotension and was given on intravenous fluids. Sepsis was suspected and broad spectrum antibiotics were initiated. The early differential diagnosis included Steven Johnson syndrome vs. necrotizing fasciitis causing sepsis. Her labs revealed severe pancytopenia and severe metabolic acidosis. Detailed results of the laboratory parameters are given in Table 1.

**Figure 2:**
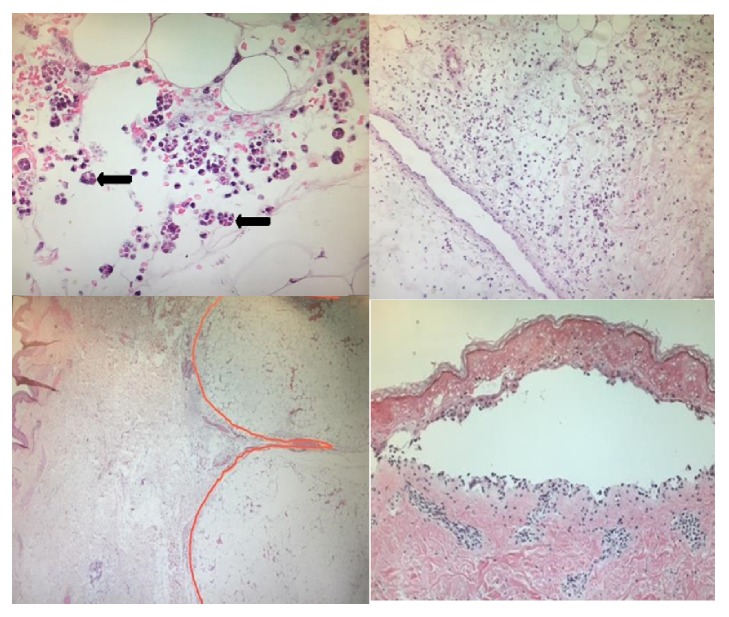
(a) The arrows indicate macrophages engulfing mostly the red blood cells (bean bag cells), (b) this is lobular panniculitis comprised of cellular infiltrate of lymphocytes, macrophages, and plasma cells, (c) septal panniculitis (highlighted), and (d) subepidermal bullous lesion.

She was transfused 2 units of packed red blood cells and 6 units of platelets. Within an hour of arrival to the ER she developed acute respiratory failure and was intubated and placed on mechanical ventilation. She developed septic shock requiring vasopressors. She was deemed too unstable for imaging studies but a portable chest X-ray revealed right basilar atelectasis and portable X-ray of the lower extremities showed soft tissue edema. Four hours after the initial presentation she developed asystole and expired after failed resuscitative measures. An autopsy was done that revealed CHP (Figure 2), Libman Sacks endocarditis, bilateral pleural effusion, proliferative and membranous lupus glomerular nephropathy, and bilateral adrenal hemorrhage. Blood culture postmortem grew serratia marcescens.

## 3. Discussion

CHP has an elaborate spectrum of clinical presentations. It can present with a wide array of symptoms ranging from local skin lesions to a life threatening systemic illness as associated with hemophagocytic syndrome (HLH) [12]. It can present with recurrent fever, subcutaneous nodules, hepatosplenomegaly, abnormal liver function tests, pancytopenia, polyserositis, and hemorrhagic diathesis [2, 13].

Its clinical course is variable depends on degree of severity. Some patients developed chronicity having recurring bouts over the course of their illness and surviving for years [14–16]. In others, it can rapidly progress resulting in the demise of the patients from sepsis and multiorgan failure [2, 13, 17]. Diagnosis is particularly challenging as it is a rare disorder and limited literature is available for guiding the management of this disease. The differential diagnosis is wide, including tuberculosis and histiocytosis. The diagnosis requires a skin biopsy, which shows characteristic histologic findings; “bean bag cells” are pathognomonic.

The current treatment options include high dose of steroids and immunosuppressants such as cyclosporine A as the first line agents [9, 18]. Recently other treatment modalities like chemotherapy have also been used successfully, CHOP (cyclophosphamide, doxorubicin, vincristine, and prednisolone) being the most commonly used chemotherapy regimen. In treatment refractory cases the use of azathioprine, cyclophosphamide, tacrolimus, plasmapheresis, and autologous peripheral blood stem cell transplantation has also been reported [19].

CHP has a poor prognosis and a high mortality up to 70% in severe cases, while other cases are successfully treated with immunosuppressive agents like cyclosporine [9, 15, 18, 19]. CHP in patients with SLE is a rare combination and to date only a handful such cases have been reported in medical literature. Fever, lymphadenopathy, and pancytopenia are some of the clinical features that indicate poor prognosis in such patients [9]. Our patient had pancytopenia and had a rapidly progressive decline in clinical picture that is characteristic of this disorder in severe cases as mentioned earlier. Unfortunately our patient expired in a very short period of presentation to the ER and diagnosis was made postmortem with autopsy. Clinicians should be aware of this uncommon disorder as the delay in treatment can be rapidly fatal.

## Figures and Tables

**Table 1:** Initial laboratory workup.

Parameter	Initial laboratory results	Reference range
Hemoglobin (g/dl)	3.9	12-16

White blood cell count (per mm^3^)	0.6	4.8-10.8

Platelet count (k/ul)	11	150-400

Sodium (mEq/L)	129	135-145

Potassium (mEq/L)	7.1	3.5-5.0

HCO3 (mEq/L)	11	24-30

Ph	6.9	7.35-7.45

BUN (mg/dl)	52	6- 20

Creatinine (mg/dl)	3	0.5 -1.5

Glucose (mg/dl)	376	70-120

Lactic acid (mmoles/L)	6.7	0.5-1.6

AST (mg/dl)	50	9-48

ALT (mg/dl)	20	5 -40

Total bilirubin/direct (mg/dl)	0.2	0.2-1.2

Alkaline phosphatase U/L	20	53-141

Albumin (g/dl)	0.4	3.2-4.8

Urine toxicology screen	negative	negative

Blood culture	Serratia marcescens positive	negative
